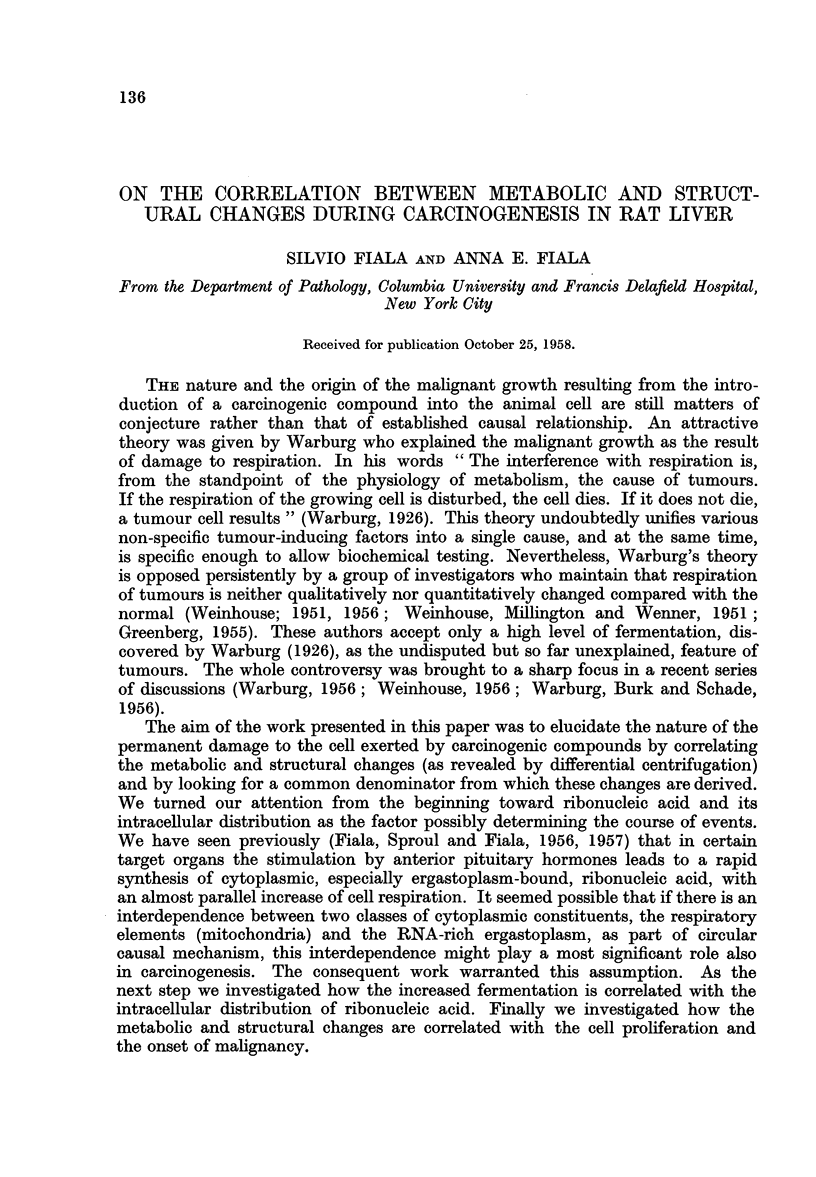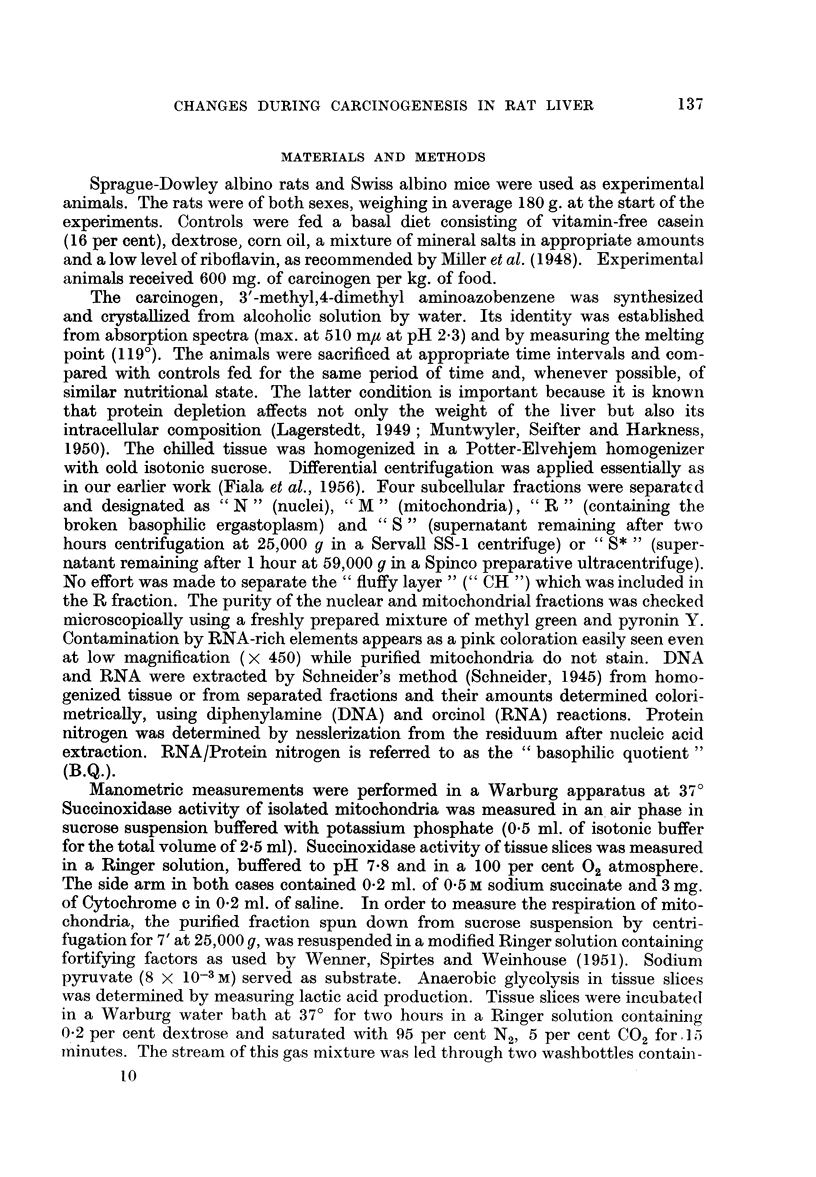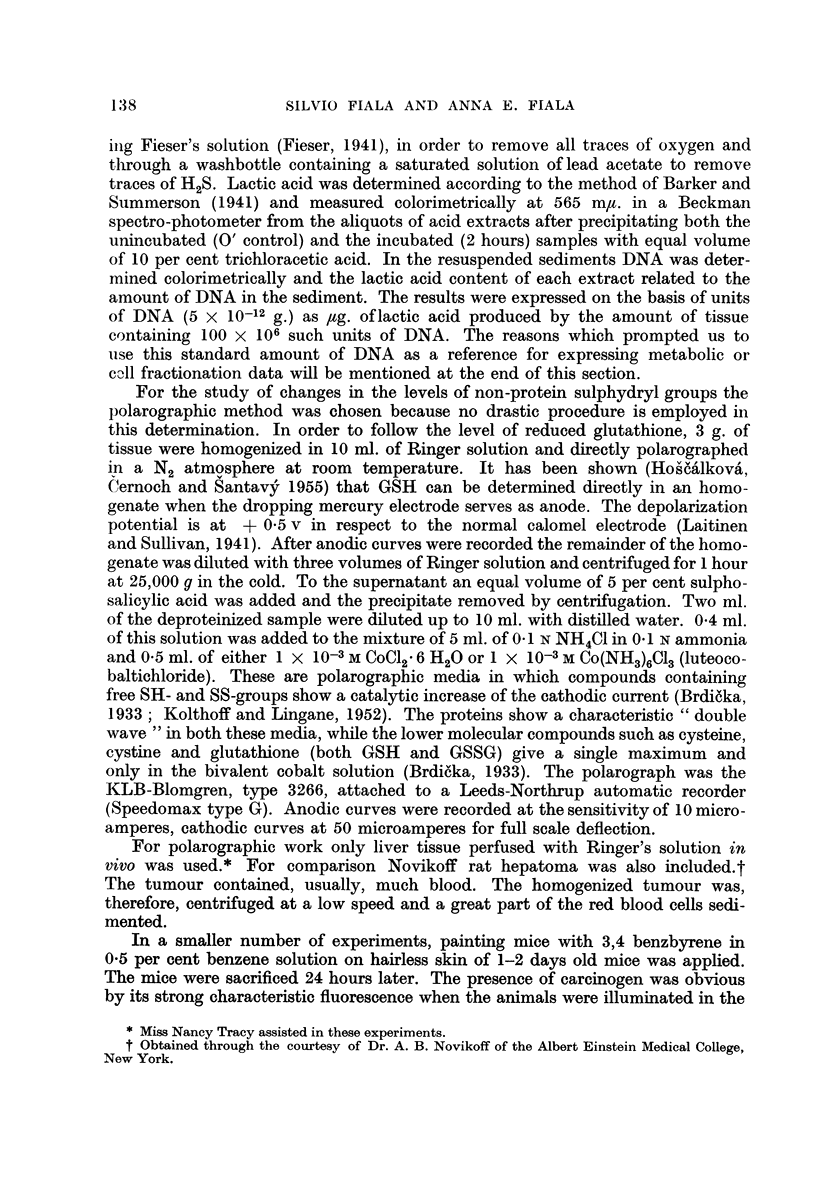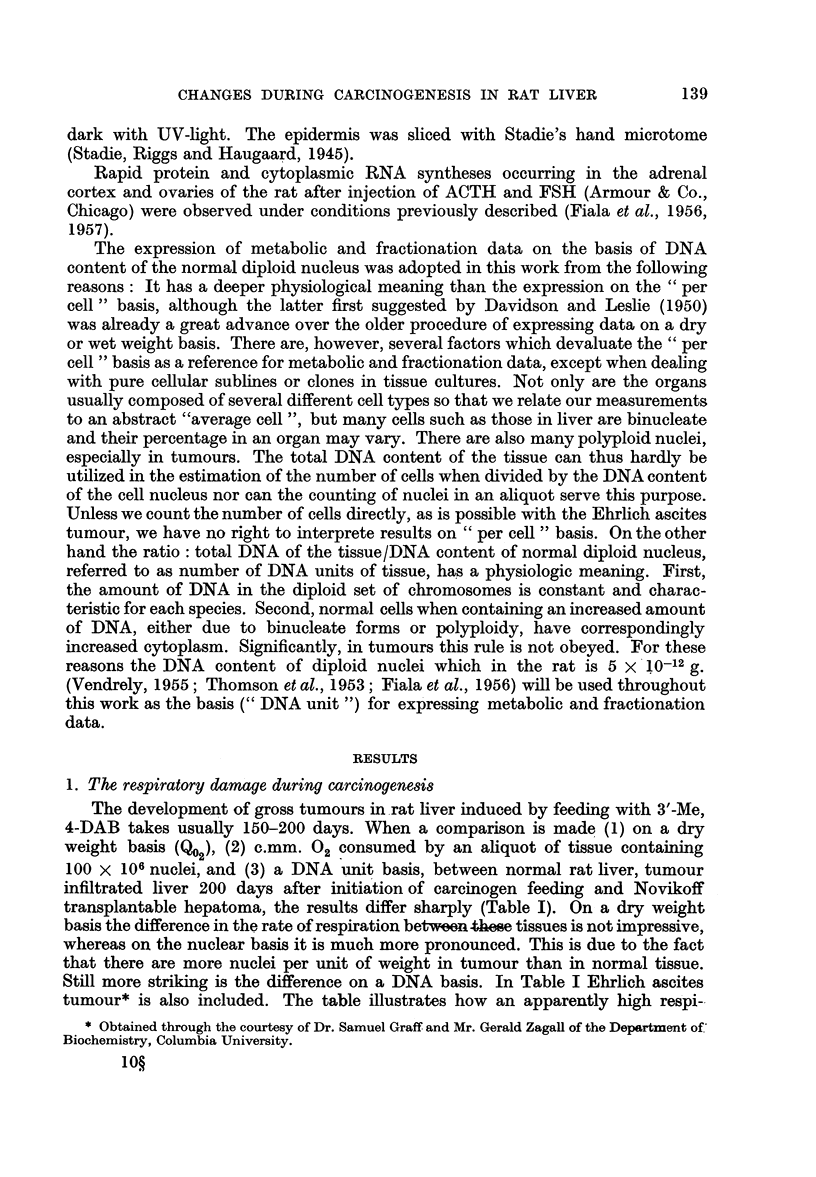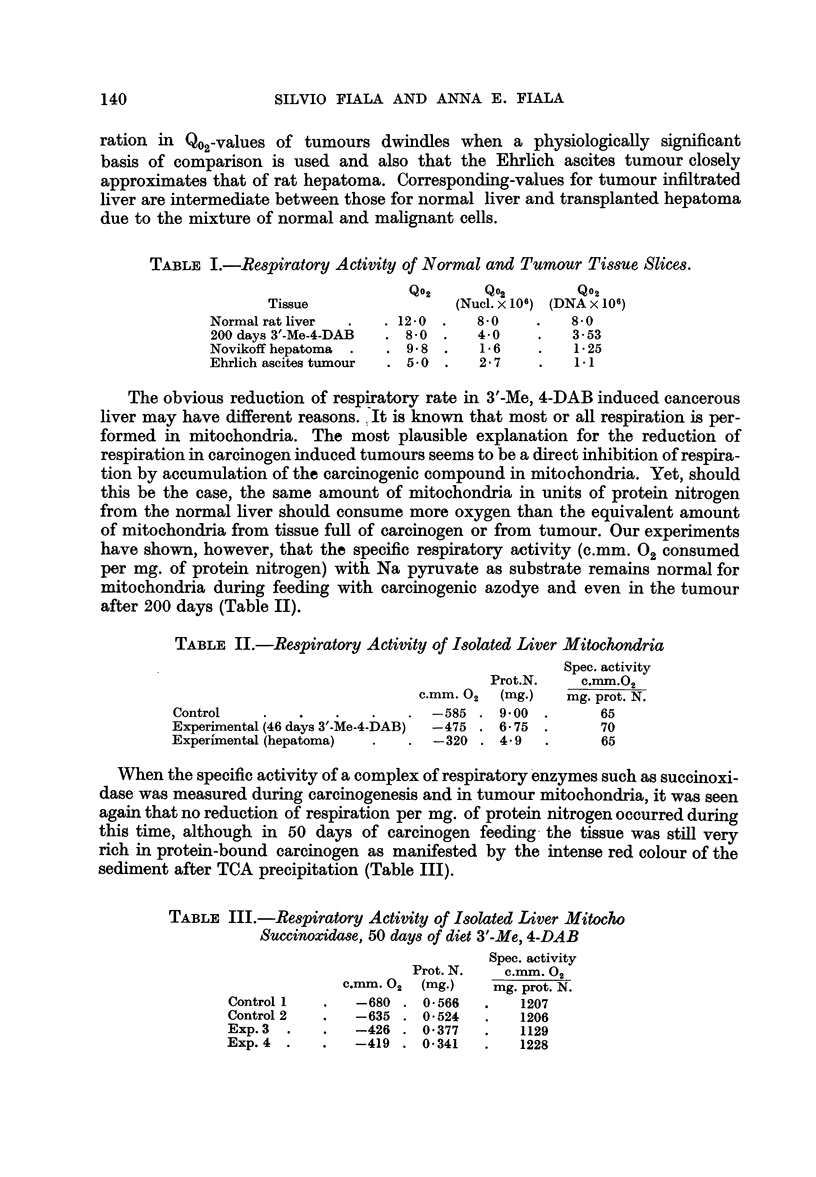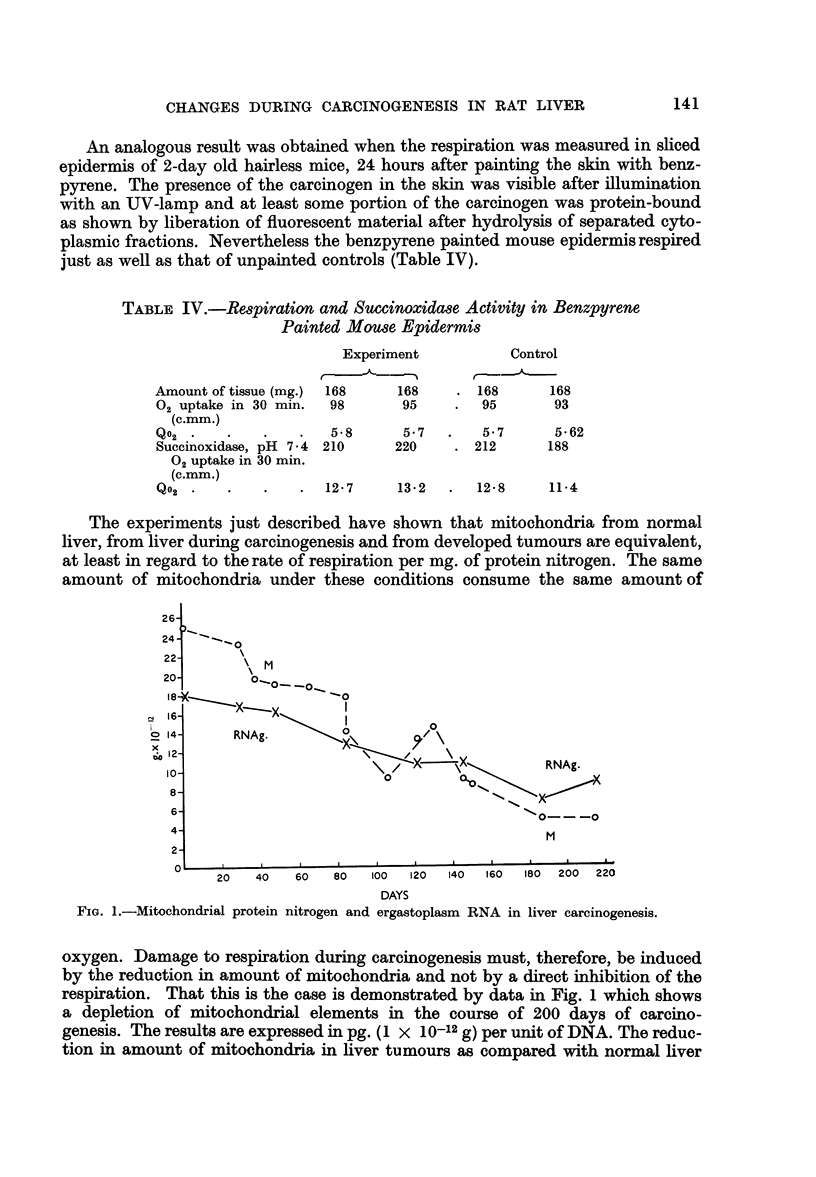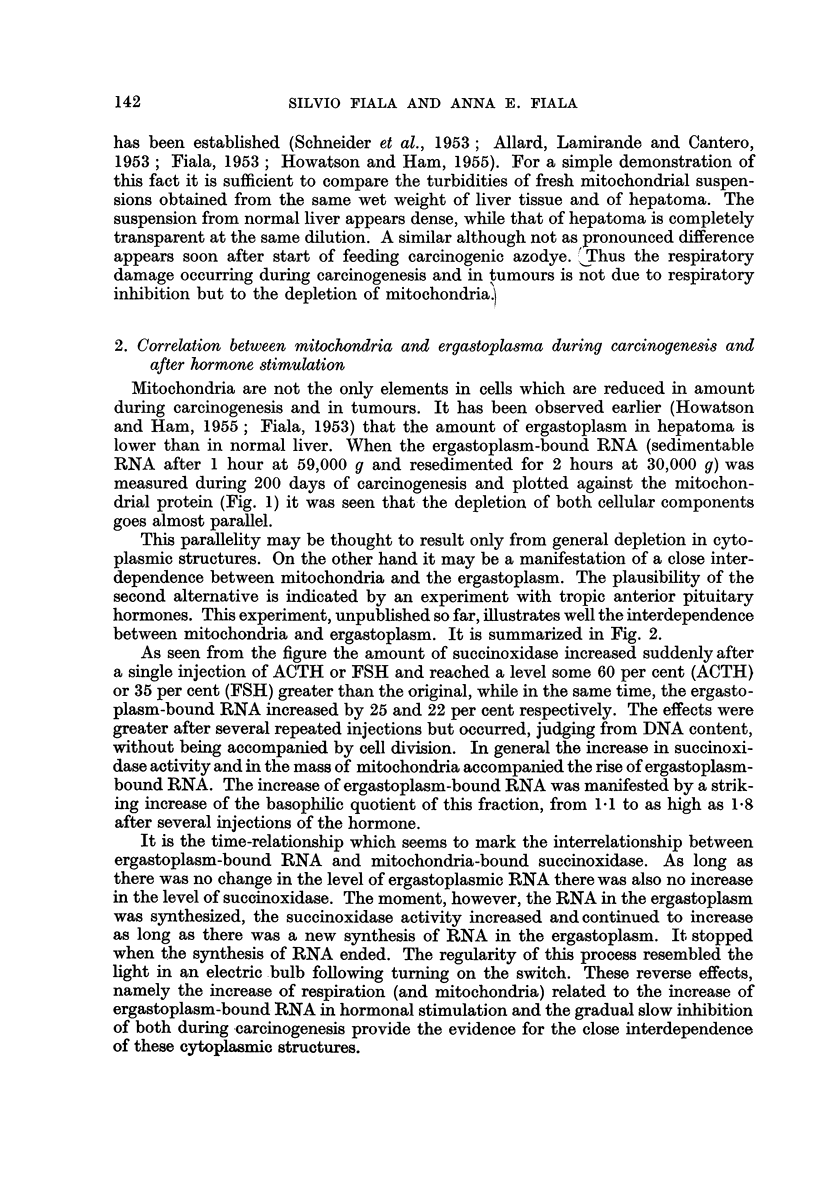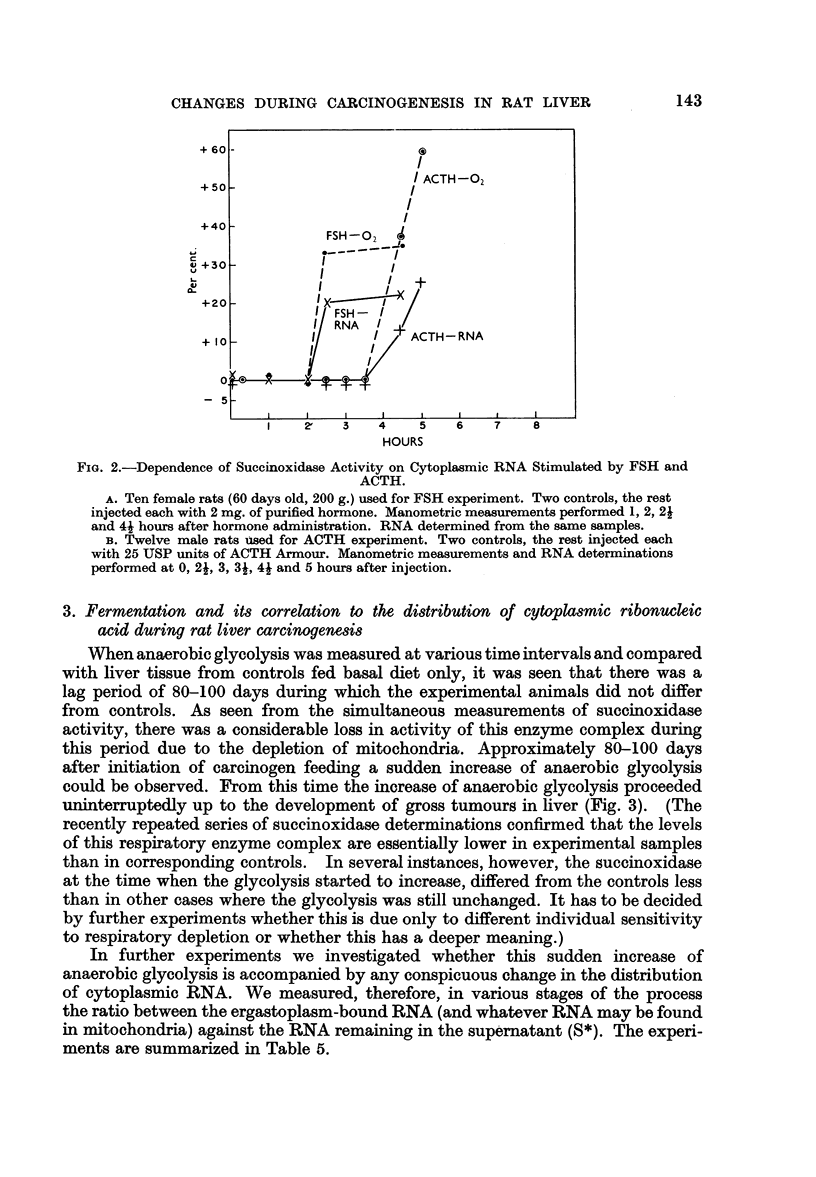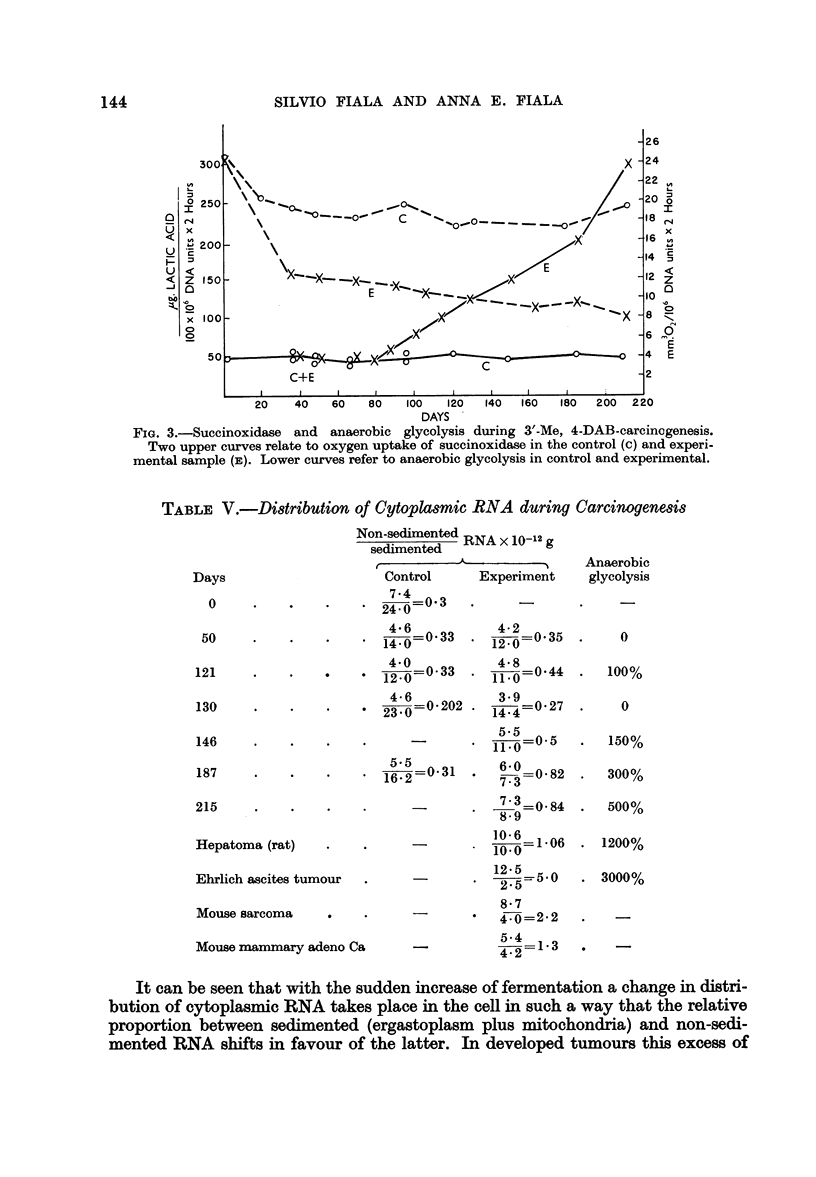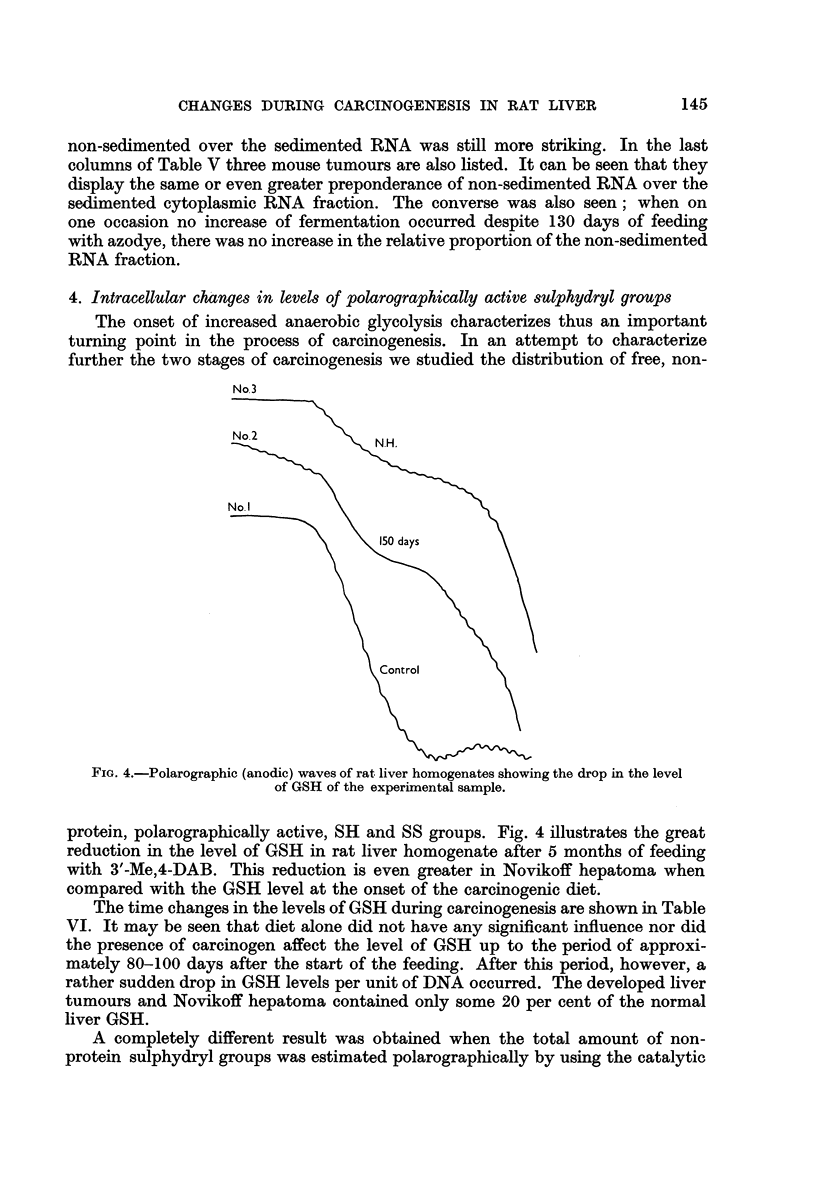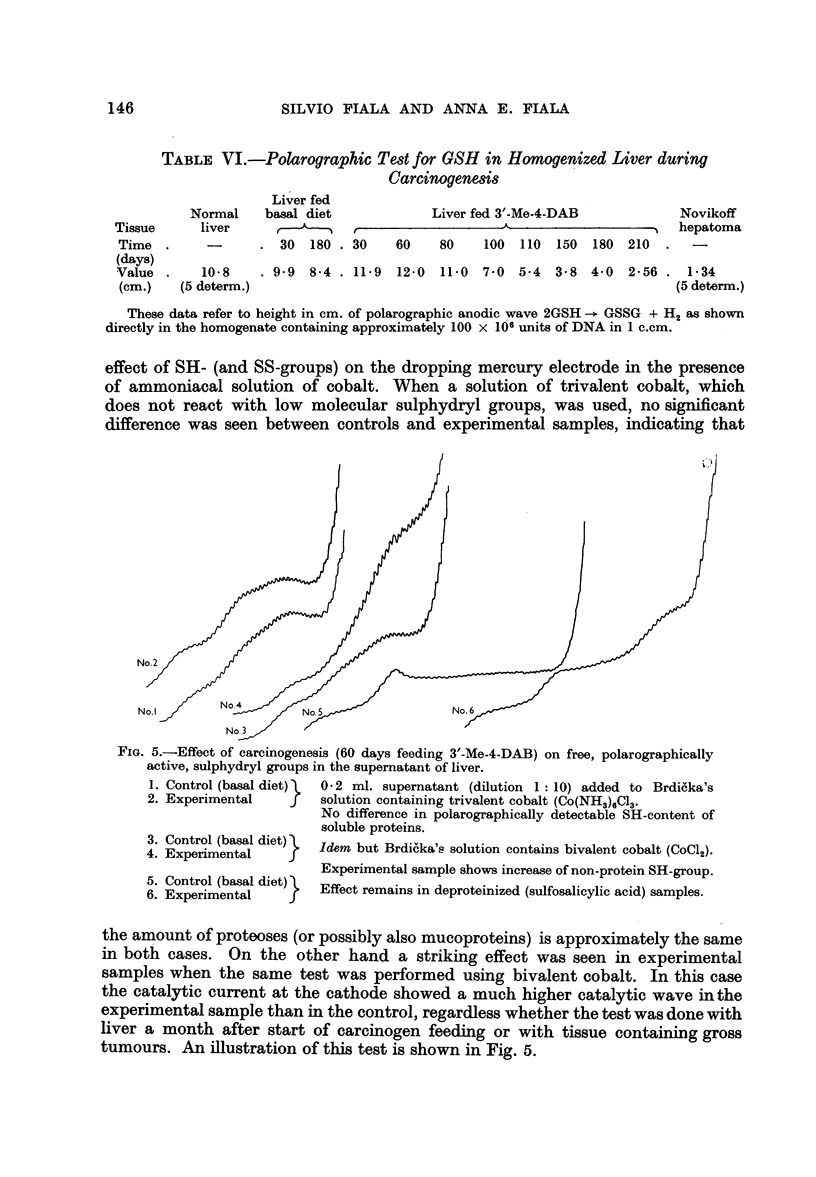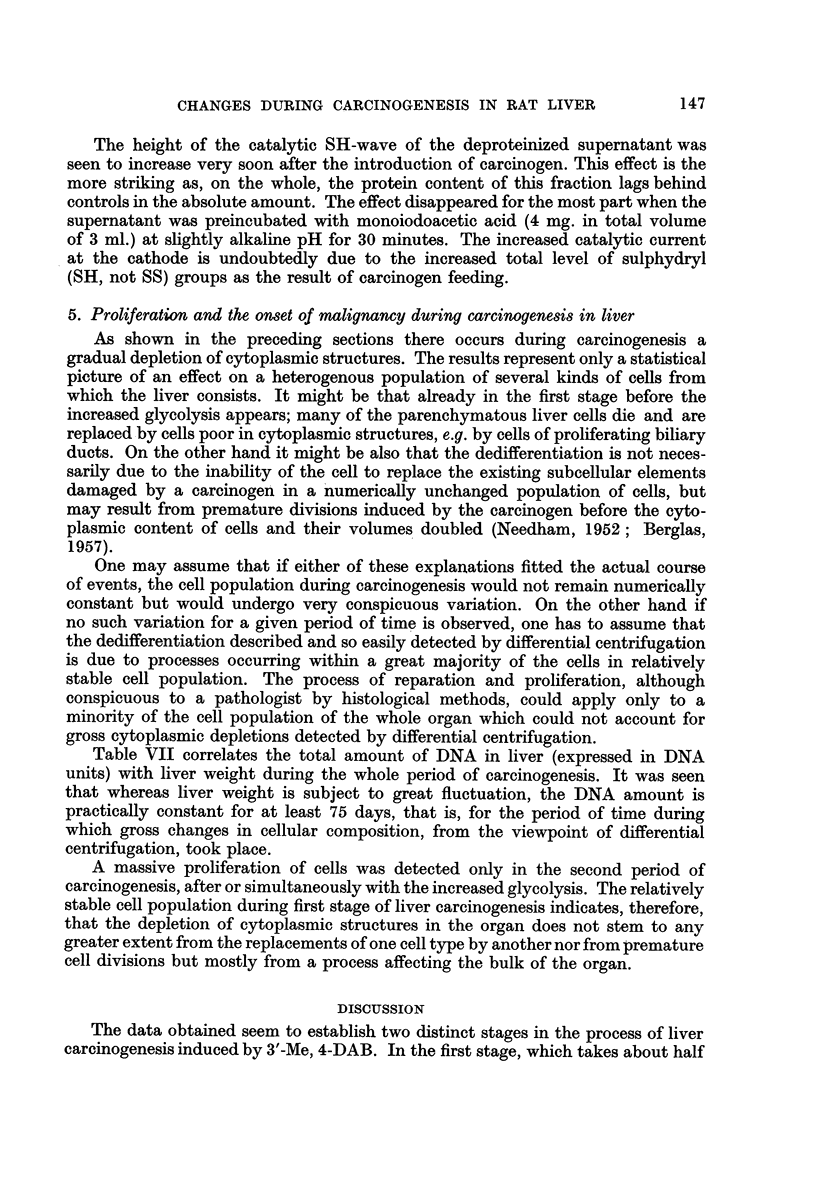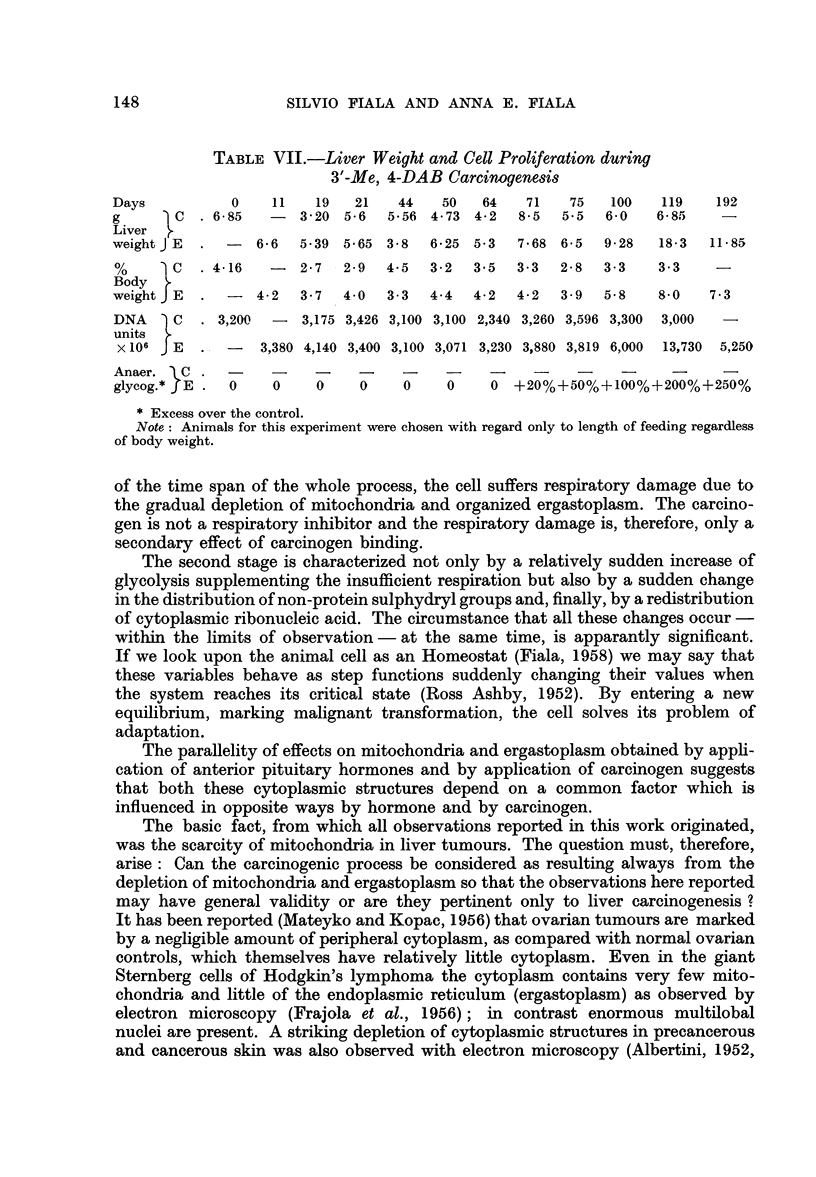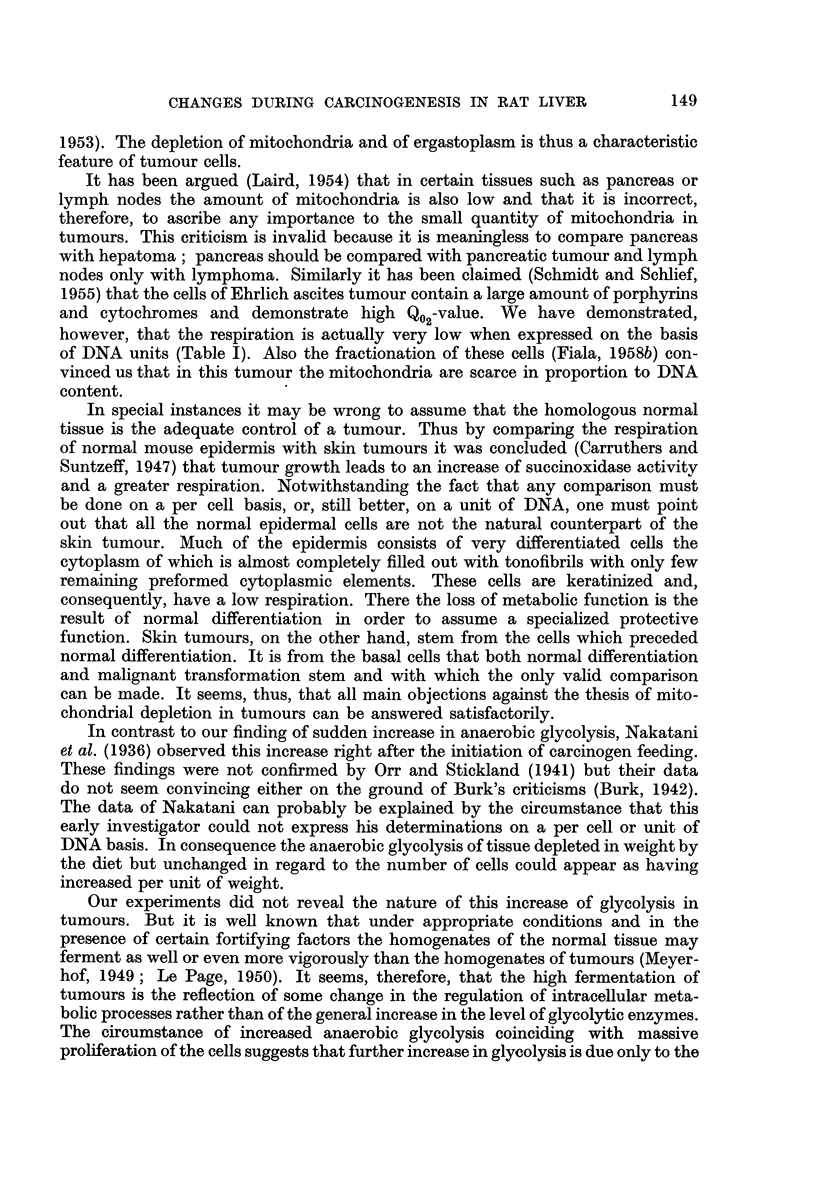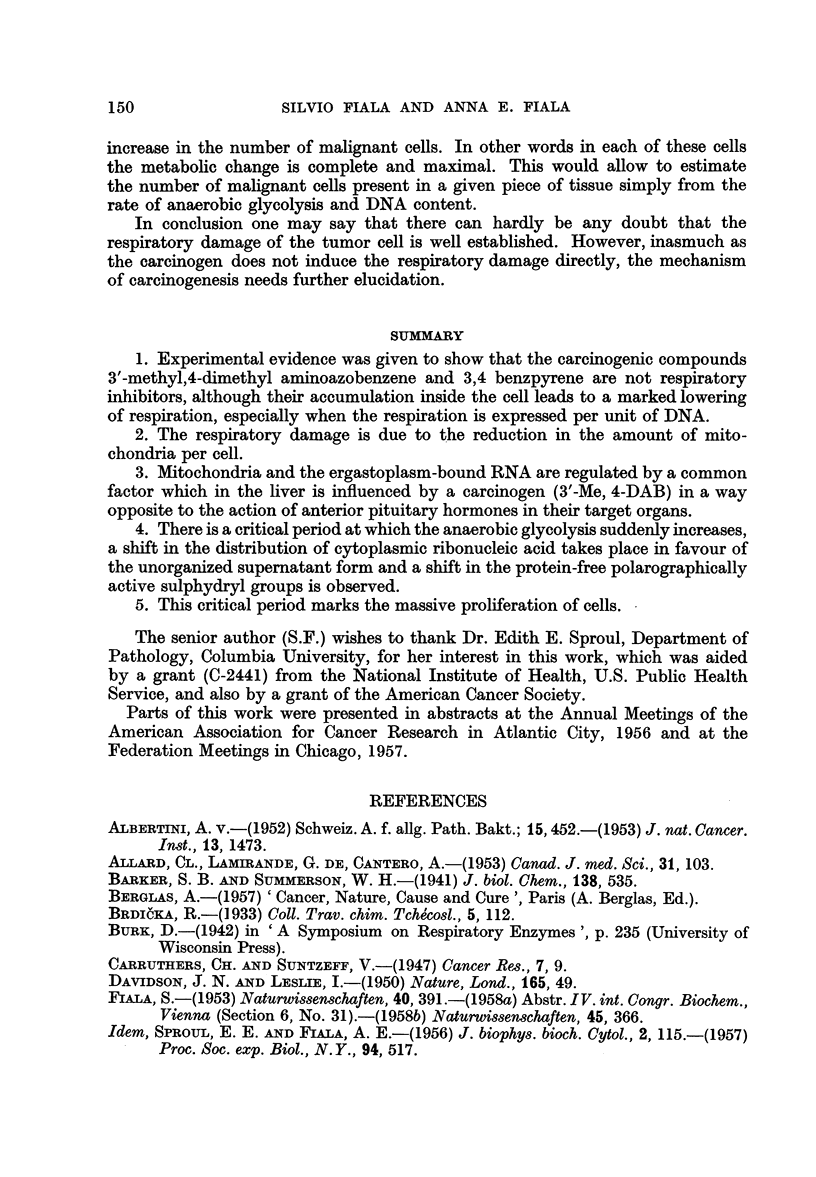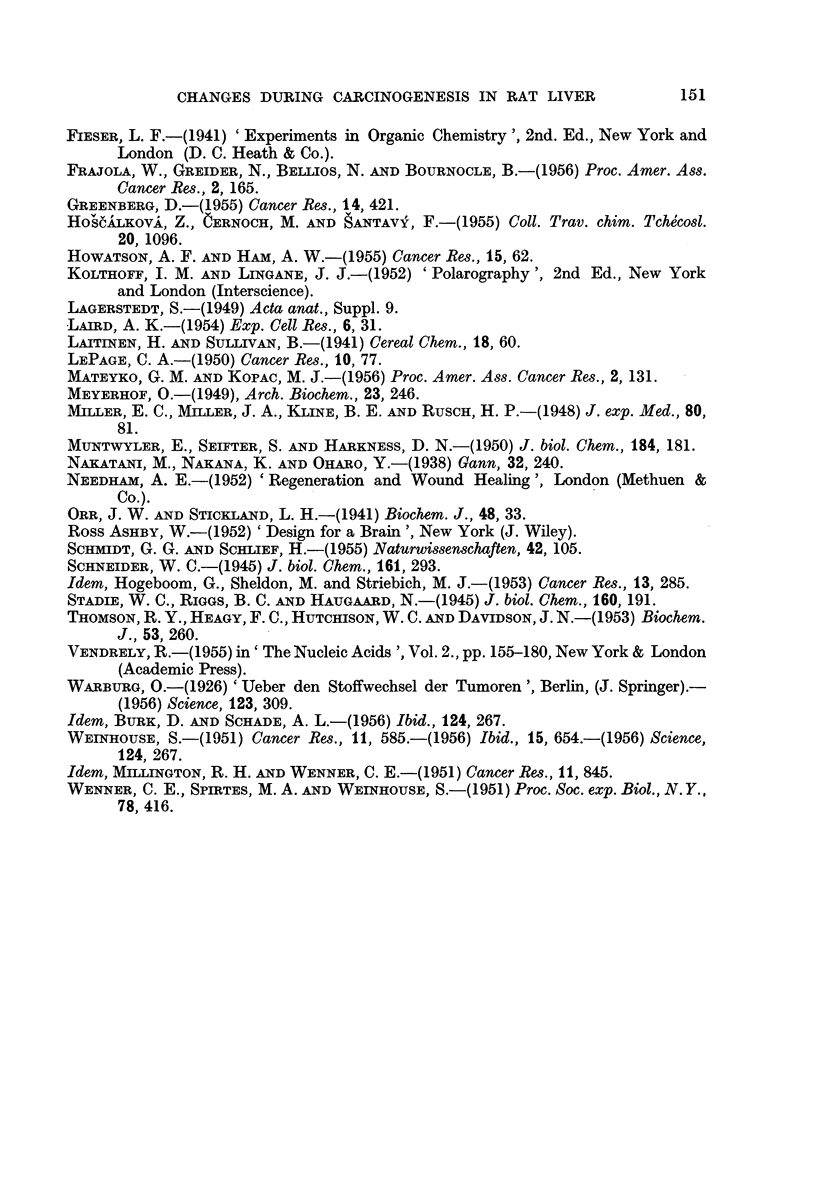# On the Correlation between Metabolic and Structural Changes During Carcinogenesis in Rat Liver

**DOI:** 10.1038/bjc.1959.20

**Published:** 1959-03

**Authors:** Silvio Fiala, Anna E. Fiala


### ON THE CORRELATION BETWEEN METABOLIC AND STRUCTURAL CHANGES DURING CARCINOGENESIS IN RAT LIVER

SILVIO FIALA AND ANNA E. FIALA

*From the Department of Pathology, Columbia University and Francis Delafield Hospital, New York City*

Received for publication October 25, 1958.

THE nature and the origin of the malignant growth resulting from the introduction of a carcinogenic compound into the animal cell are still matters of conjecture rather than that of established causal relationship. An attractive theory was given by Warburg who explained the malignant growth as the result of damage to respiration. In his words " The interference with respiration is, from the standpoint of the physiology of metabolism, the cause of tumours. If the respiration of the growing cell is disturbed, the cell dies. If it does not die, a tumour cell results " (Warburg, 1926). This theory undoubtedly unifies various non-specific tumour-inducing factors into a single cause, and at the same time, is specific enough to allow biochemical testing. Nevertheless, Warburg's theory is opposed persistently by a group of investigators who maintain that respiration of tumours is neither qualitatively nor quantitatively changed compared with the normal (Weinhouse; 1951, 1956 ; Weinhouse, Millington and Wenner, 1951 ; Greenberg, 1955). These authors accept only a high level of fermentation, discovered by Warburg (1926), as the undisputed but so far unexplained, feature of tumours. The whole controversy was brought to a sharp focus in a recent series of discussions (Warburg, 1956 ; Weinhouse, 1956 ; Warburg, Burk and Schade, 1956).

The aim of the work presented in this paper was to elucidate the nature of the permanent damage to the cell exerted by carcinogenic compounds by correlating the metabolic and structural changes (as revealed by differential centrifugation) and by looking for a common denominator from which these changes are derived. We turned our attention from the beginning toward ribonucleic acid and its intracellular distribution as the factor possibly determining the course of events. We have seen previously (Fiala, Sproul and Fiala, 1956, 1957) that in certain target organs the stimulation by anterior pituitary hormones leads to a rapid synthesis of cytoplasmic, especially ergastoplasm-bound, ribonucleic acid, with an almost parallel increase of cell respiration. It seemed possible that if there is an interdependence between two classes of cytoplasmic constituents, the respiratory elements (mitochondria) and the RNA-rich ergastoplasm, as part of circular causal mechanism, this interdependence might play a most significant role also in carcinogenesis. The consequent work warranted this assumption. As the next step we investigated how the increased fermentation is correlated with the intracellular distribution of ribonucleic acid. Finally we investigated how the metabolic and structural changes are correlated with the cell proliferation and the onset of malignancy.

### MATERIALS AND METHODS

Sprague-Dowley albino rats and Swiss albino mice were used as experimental animals. The rats were of both sexes, weighing in average 180 g. at the start of the experiments. Controls were fed a basal diet consisting of vitamin-free casein (16 per cent), dextrose, corn oil, a mixture of mineral salts in appropriate amounts and a low level of riboflavin, as recommended by Miller *et al.* (1948). Experimental animals received 600 mg. of carcinogen per kg. of food.

The carcinogen, 3′-methyl,4-dimethyl aminoazobenzene was synthesized and crystallized from alcoholic solution by water. Its identity was established from absorption spectra (max. at 510 m$\mu$ at pH 2·3) and by measuring the melting point (119°). The animals were sacrificed at appropriate time intervals and compared with controls fed for the same period of time and, whenever possible, of similar nutritional state. The latter condition is important because it is known that protein depletion affects not only the weight of the liver but also its intracellular composition (Lagerstedt, 1949 ; Muntwyler, Seifter and Harkness, 1950). The chilled tissue was homogenized in a Potter-Elvehjem homogenizer with cold isotonic sucrose. Differential centrifugation was applied essentially as in our earlier work (Fiala *et al.*, 1956). Four subcellular fractions were separated and designated as " N " (nuclei), " M " (mitochondria), " R " (containing the broken basophilic ergastoplasm) and " S " (supernatant remaining after two hours centrifugation at 25,000 *g* in a Servall SS-1 centrifuge) or " S* " (supernatant remaining after 1 hour at 59,000 *g* in a Spinco preparative ultracentrifuge). No effort was made to separate the " fluffy layer " (" CH ") which was included in the R fraction. The purity of the nuclear and mitochondrial fractions was checked microscopically using a freshly prepared mixture of methyl green and pyronin Y. Contamination by RNA-rich elements appears as a pink coloration easily seen even at low magnification (× 450) while purified mitochondria do not stain. DNA and RNA were extracted by Schneider's method (Schneider, 1945) from homogenized tissue or from separated fractions and their amounts determined colorimetrically, using diphenylamine (DNA) and orcinol (RNA) reactions. Protein nitrogen was determined by nesslerization from the residuum after nucleic acid extraction. RNA/Protein nitrogen is referred to as the " basophilic quotient " (B.Q.).

Manometric measurements were performed in a Warburg apparatus at 37° Succinoxidase activity of isolated mitochondria was measured in an air phase in sucrose suspension buffered with potassium phosphate (0·5 ml. of isotonic buffer for the total volume of 2·5 ml). Succinoxidase activity of tissue slices was measured in a Ringer solution, buffered to pH 7·8 and in a 100 per cent $O_2$ atmosphere. The side arm in both cases contained 0·2 ml. of 0·5 M sodium succinate and 3 mg. of Cytochrome c in 0·2 ml. of saline. In order to measure the respiration of mitochondria, the purified fraction spun down from sucrose suspension by centrifugation for 7′ at 25,000 *g*, was resuspended in a modified Ringer solution containing fortifying factors as used by Wenner, Spirtes and Weinhouse (1951). Sodium pyruvate ($8 \times 10^{-3}$ M) served as substrate. Anaerobic glycolysis in tissue slices was determined by measuring lactic acid production. Tissue slices were incubated in a Warburg water bath at 37° for two hours in a Ringer solution containing 0·2 per cent dextrose and saturated with 95 per cent $N_2$, 5 per cent $CO_2$ for 15 minutes. The stream of this gas mixture was led through two washbottles containing Fieser's solution (Fieser, 1941), in order to remove all traces of oxygen and through a washbottle containing a saturated solution of lead acetate to remove traces of $H_2S$. Lactic acid was determined according to the method of Barker and Summerson (1941) and measured colorimetrically at 565 m$\mu$. in a Beckman spectro-photometer from the aliquots of acid extracts after precipitating both the unincubated (O' control) and the incubated (2 hours) samples with equal volume of 10 per cent trichloracetic acid. In the resuspended sediments DNA was determined colorimetrically and the lactic acid content of each extract related to the amount of DNA in the sediment. The results were expressed on the basis of units of DNA ($5 \times 10^{-12}$ g.) as $\mu$g. oflactic acid produced by the amount of tissue containing $100 \times 10^6$ such units of DNA. The reasons which prompted us to use this standard amount of DNA as a reference for expressing metabolic or cell fractionation data will be mentioned at the end of this section.

For the study of changes in the levels of non-protein sulphydryl groups the polarographic method was chosen because no drastic procedure is employed in this determination. In order to follow the level of reduced glutathione, 3 g. of tissue were homogenized in 10 ml. of Ringer solution and directly polarographed in a $N_2$ atmosphere at room temperature. It has been shown (Hoščálková, Černoch and Šantavý 1955) that GSH can be determined directly in an homogenate when the dropping mercury electrode serves as anode. The depolarization potential is at + 0·5 v in respect to the normal calomel electrode (Laitinen and Sullivan, 1941). After anodic curves were recorded the remainder of the homogenate was diluted with three volumes of Ringer solution and centrifuged for 1 hour at 25,000 *g* in the cold. To the supernatant an equal volume of 5 per cent sulphosalicylic acid was added and the precipitate removed by centrifugation. Two ml. of the deproteinized sample were diluted up to 10 ml. with distilled water. 0·4 ml. of this solution was added to the mixture of 5 ml. of 0·1 N $NH_4Cl$ in 0·1 N ammonia and 0·5 ml. of either $1 \times 10^{-3}$ M $CoCl_2 \cdot 6\,H_2O$ or $1 \times 10^{-3}$ M $Co(NH_3)_6Cl_3$ (luteocobaltichloride). These are polarographic media in which compounds containing free SH- and SS-groups show a catalytic increase of the cathodic current (Brdička, 1933; Kolthoff and Lingane, 1952). The proteins show a characteristic " double wave " in both these media, while the lower molecular compounds such as cysteine, cystine and glutathione (both GSH and GSSG) give a single maximum and only in the bivalent cobalt solution (Brdička, 1933). The polarograph was the KLB-Blomgren, type 3266, attached to a Leeds-Northrup automatic recorder (Speedomax type G). Anodic curves were recorded at the sensitivity of 10 microamperes, cathodic curves at 50 microamperes for full scale deflection.

For polarographic work only liver tissue perfused with Ringer's solution *in vivo* was used.* For comparison Novikoff rat hepatoma was also included.† The tumour contained, usually, much blood. The homogenized tumour was, therefore, centrifuged at a low speed and a great part of the red blood cells sedimented.

In a smaller number of experiments, painting mice with 3,4 benzbyrene in 0·5 per cent benzene solution on hairless skin of 1–2 days old mice was applied. The mice were sacrificed 24 hours later. The presence of carcinogen was obvious by its strong characteristic fluorescence when the animals were illuminated in the

* Miss Nancy Tracy assisted in these experiments.
† Obtained through the courtesy of Dr. A. B. Novikoff of the Albert Einstein Medical College, New York.

dark with UV-light. The epidermis was sliced with Stadie's hand microtome (Stadie, Riggs and Haugaard, 1945).

Rapid protein and cytoplasmic RNA syntheses occurring in the adrenal cortex and ovaries of the rat after injection of ACTH and FSH (Armour & Co., Chicago) were observed under conditions previously described (Fiala *et al.*, 1956, 1957).

The expression of metabolic and fractionation data on the basis of DNA content of the normal diploid nucleus was adopted in this work from the following reasons : It has a deeper physiological meaning than the expression on the " per cell " basis, although the latter first suggested by Davidson and Leslie (1950) was already a great advance over the older procedure of expressing data on a dry or wet weight basis. There are, however, several factors which devaluate the " per cell " basis as a reference for metabolic and fractionation data, except when dealing with pure cellular sublines or clones in tissue cultures. Not only are the organs usually composed of several different cell types so that we relate our measurements to an abstract "average cell ", but many cells such as those in liver are binucleate and their percentage in an organ may vary. There are also many polyploid nuclei, especially in tumours. The total DNA content of the tissue can thus hardly be utilized in the estimation of the number of cells when divided by the DNA content of the cell nucleus nor can the counting of nuclei in an aliquot serve this purpose. Unless we count the number of cells directly, as is possible with the Ehrlich ascites tumour, we have no right to interprete results on " per cell " basis. On the other hand the ratio : total DNA of the tissue/DNA content of normal diploid nucleus, referred to as number of DNA units of tissue, has a physiologic meaning. First, the amount of DNA in the diploid set of chromosomes is constant and characteristic for each species. Second, normal cells when containing an increased amount of DNA, either due to binucleate forms or polyploidy, have correspondingly increased cytoplasm. Significantly, in tumours this rule is not obeyed. For these reasons the DNA content of diploid nuclei which in the rat is $5 \times 10^{-12}$ g. (Vendrely, 1955 ; Thomson *et al.*, 1953 ; Fiala *et al.*, 1956) will be used throughout this work as the basis (" DNA unit ") for expressing metabolic and fractionation data.

### RESULTS

### 1. *The respiratory damage during carcinogenesis*

The development of gross tumours in rat liver induced by feeding with 3′-Me, 4-DAB takes usually 150–200 days. When a comparison is made (1) on a dry weight basis ($Q_{O_2}$), (2) c.mm. $O_2$ consumed by an aliquot of tissue containing $100 \times 10^6$ nuclei, and (3) a DNA unit basis, between normal rat liver, tumour infiltrated liver 200 days after initiation of carcinogen feeding and Novikoff transplantable hepatoma, the results differ sharply (Table I). On a dry weight basis the difference in the rate of respiration between these tissues is not impressive, whereas on the nuclear basis it is much more pronounced. This is due to the fact that there are more nuclei per unit of weight in tumour than in normal tissue. Still more striking is the difference on a DNA basis. In Table I Ehrlich ascites tumour* is also included. The table illustrates how an apparently high respi-

* Obtained through the courtesy of Dr. Samuel Graff and Mr. Gerald Zagall of the Department of Biochemistry, Columbia University.

ration in $Q_{O_2}$-values of tumours dwindles when a physiologically significant basis of comparison is used and also that the Ehrlich ascites tumour closely approximates that of rat hepatoma. Corresponding-values for tumour infiltrated liver are intermediate between those for normal liver and transplanted hepatoma due to the mixture of normal and malignant cells.

TABLE I.—*Respiratory Activity of Normal and Tumour Tissue Slices.*

| Tissue | $Q_{O_2}$ | $Q_{O_2}$ (Nucl. $\times 10^6$) | $Q_{O_2}$ (DNA $\times 10^6$) |
|---|---|---|---|
| Normal rat liver | 12·0 | 8·0 | 8·0 |
| 200 days 3′-Me-4-DAB | 8·0 | 4·0 | 3·53 |
| Novikoff hepatoma | 9·8 | 1·6 | 1·25 |
| Ehrlich ascites tumour | 5·0 | 2·7 | 1·1 |

The obvious reduction of respiratory rate in 3′-Me, 4-DAB induced cancerous liver may have different reasons. It is known that most or all respiration is performed in mitochondria. The most plausible explanation for the reduction of respiration in carcinogen induced tumours seems to be a direct inhibition of respiration by accumulation of the carcinogenic compound in mitochondria. Yet, should this be the case, the same amount of mitochondria in units of protein nitrogen from the normal liver should consume more oxygen than the equivalent amount of mitochondria from tissue full of carcinogen or from tumour. Our experiments have shown, however, that the specific respiratory activity (c.mm. $O_2$ consumed per mg. of protein nitrogen) with Na pyruvate as substrate remains normal for mitochondria during feeding with carcinogenic azodye and even in the tumour after 200 days (Table II).

TABLE II.—*Respiratory Activity of Isolated Liver Mitochondria*

| | c.mm. $O_2$ | Prot.N. (mg.) | Spec. activity $\frac{\text{c.mm.} O_2}{\text{mg. prot. N.}}$ |
|---|---|---|---|
| Control | −585 | 9·00 | 65 |
| Experimental (46 days 3′-Me-4-DAB) | −475 | 6·75 | 70 |
| Experimental (hepatoma) | −320 | 4·9 | 65 |

When the specific activity of a complex of respiratory enzymes such as succinoxidase was measured during carcinogenesis and in tumour mitochondria, it was seen again that no reduction of respiration per mg. of protein nitrogen occurred during this time, although in 50 days of carcinogen feeding the tissue was still very rich in protein-bound carcinogen as manifested by the intense red colour of the sediment after TCA precipitation (Table III).

TABLE III.—*Respiratory Activity of Isolated Liver Mitocho Succinoxidase, 50 days of diet 3′-Me, 4-DAB*

| | c.mm. $O_2$ | Prot. N. (mg.) | Spec. activity $\frac{\text{c.mm.} O_2}{\text{mg. prot. N.}}$ |
|---|---|---|---|
| Control 1 | −680 | 0·566 | 1207 |
| Control 2 | −635 | 0·524 | 1206 |
| Exp. 3 | −426 | 0·377 | 1129 |
| Exp. 4 | −419 | 0·341 | 1228 |

An analogous result was obtained when the respiration was measured in sliced epidermis of 2-day old hairless mice, 24 hours after painting the skin with benzpyrene. The presence of the carcinogen in the skin was visible after illumination with an UV-lamp and at least some portion of the carcinogen was protein-bound as shown by liberation of fluorescent material after hydrolysis of separated cytoplasmic fractions. Nevertheless the benzpyrene painted mouse epidermis respired just as well as that of unpainted controls (Table IV).

TABLE IV.—*Respiration and Succinoxidase Activity in Benzpyrene Painted Mouse Epidermis*

| | Experiment | | Control | |
|---|---|---|---|---|
| Amount of tissue (mg.) | 168 | 168 | 168 | 168 |
| $O_2$ uptake in 30 min. (c.mm.) | 98 | 95 | 95 | 93 |
| $Q_{O_2}$ | 5·8 | 5·7 | 5·7 | 5·62 |
| Succinoxidase, pH 7·4 $O_2$ uptake in 30 min. (c.mm.) | 210 | 220 | 212 | 188 |
| $Q_{O_2}$ | 12·7 | 13·2 | 12·8 | 11·4 |

The experiments just described have shown that mitochondria from normal liver, from liver during carcinogenesis and from developed tumours are equivalent, at least in regard to the rate of respiration per mg. of protein nitrogen. The same amount of mitochondria under these conditions consume the same amount of

FIG. 1.—Mitochondrial protein nitrogen and ergastoplasm RNA in liver carcinogenesis.

oxygen. Damage to respiration during carcinogenesis must, therefore, be induced by the reduction in amount of mitochondria and not by a direct inhibition of the respiration. That this is the case is demonstrated by data in Fig. 1 which shows a depletion of mitochondrial elements in the course of 200 days of carcinogenesis. The results are expressed in pg. ($1 \times 10^{-12}$ g) per unit of DNA. The reduction in amount of mitochondria in liver tumours as compared with normal liver has been established (Schneider *et al.*, 1953 ; Allard, Lamirande and Cantero, 1953 ; Fiala, 1953 ; Howatson and Ham, 1955). For a simple demonstration of this fact it is sufficient to compare the turbidities of fresh mitochondrial suspensions obtained from the same wet weight of liver tissue and of hepatoma. The suspension from normal liver appears dense, while that of hepatoma is completely transparent at the same dilution. A similar although not as pronounced difference appears soon after start of feeding carcinogenic azodye. Thus the respiratory damage occurring during carcinogenesis and in tumours is not due to respiratory inhibition but to the depletion of mitochondria.

2. *Correlation between mitochondria and ergastoplasma during carcinogenesis and after hormone stimulation*

Mitochondria are not the only elements in cells which are reduced in amount during carcinogenesis and in tumours. It has been observed earlier (Howatson and Ham, 1955 ; Fiala, 1953) that the amount of ergastoplasm in hepatoma is lower than in normal liver. When the ergastoplasm-bound RNA (sedimentable RNA after 1 hour at 59,000 *g* and resedimented for 2 hours at 30,000 *g*) was measured during 200 days of carcinogenesis and plotted against the mitochondrial protein (Fig. 1) it was seen that the depletion of both cellular components goes almost parallel.

This parallelity may be thought to result only from general depletion in cytoplasmic structures. On the other hand it may be a manifestation of a close interdependence between mitochondria and the ergastoplasm. The plausibility of the second alternative is indicated by an experiment with tropic anterior pituitary hormones. This experiment, unpublished so far, illustrates well the interdependence between mitochondria and ergastoplasm. It is summarized in Fig. 2.

As seen from the figure the amount of succinoxidase increased suddenly after a single injection of ACTH or FSH and reached a level some 60 per cent (ACTH) or 35 per cent (FSH) greater than the original, while in the same time, the ergastoplasm-bound RNA increased by 25 and 22 per cent respectively. The effects were greater after several repeated injections but occurred, judging from DNA content, without being accompanied by cell division. In general the increase in succinoxidase activity and in the mass of mitochondria accompanied the rise of ergastoplasm-bound RNA. The increase of ergastoplasm-bound RNA was manifested by a striking increase of the basophilic quotient of this fraction, from 1·1 to as high as 1·8 after several injections of the hormone.

It is the time-relationship which seems to mark the interrelationship between ergastoplasm-bound RNA and mitochondria-bound succinoxidase. As long as there was no change in the level of ergastoplasmic RNA there was also no increase in the level of succinoxidase. The moment, however, the RNA in the ergastoplasm was synthesized, the succinoxidase activity increased and continued to increase as long as there was a new synthesis of RNA in the ergastoplasm. It stopped when the synthesis of RNA ended. The regularity of this process resembled the light in an electric bulb following turning on the switch. These reverse effects, namely the increase of respiration (and mitochondria) related to the increase of ergastoplasm-bound RNA in hormonal stimulation and the gradual slow inhibition of both during carcinogenesis provide the evidence for the close interdependence of these cytoplasmic structures.

FIG. 2.—Dependence of Succinoxidase Activity on Cytoplasmic RNA Stimulated by FSH and ACTH.

A. Ten female rats (60 days old, 200 g.) used for FSH experiment. Two controls, the rest injected each with 2 mg. of purified hormone. Manometric measurements performed 1, 2, 2½ and 4½ hours after hormone administration. RNA determined from the same samples.

B. Twelve male rats used for ACTH experiment. Two controls, the rest injected each with 25 USP units of ACTH Armour. Manometric measurements and RNA determinations performed at 0, 2½, 3, 3½, 4½ and 5 hours after injection.

### 3. *Fermentation and its correlation to the distribution of cytoplasmic ribonucleic acid during rat liver carcinogenesis*

When anaerobic glycolysis was measured at various time intervals and compared with liver tissue from controls fed basal diet only, it was seen that there was a lag period of 80–100 days during which the experimental animals did not differ from controls. As seen from the simultaneous measurements of succinoxidase activity, there was a considerable loss in activity of this enzyme complex during this period due to the depletion of mitochondria. Approximately 80–100 days after initiation of carcinogen feeding a sudden increase of anaerobic glycolysis could be observed. From this time the increase of anaerobic glycolysis proceeded uninterruptedly up to the development of gross tumours in liver (Fig. 3). (The recently repeated series of succinoxidase determinations confirmed that the levels of this respiratory enzyme complex are essentially lower in experimental samples than in corresponding controls. In several instances, however, the succinoxidase at the time when the glycolysis started to increase, differed from the controls less than in other cases where the glycolysis was still unchanged. It has to be decided by further experiments whether this is due only to different individual sensitivity to respiratory depletion or whether this has a deeper meaning.)

In further experiments we investigated whether this sudden increase of anaerobic glycolysis is accompanied by any conspicuous change in the distribution of cytoplasmic RNA. We measured, therefore, in various stages of the process the ratio between the ergastoplasm-bound RNA (and whatever RNA may be found in mitochondria) against the RNA remaining in the supernatant (S*). The experiments are summarized in Table 5.

FIG. 3.—Succinoxidase and anaerobic glycolysis during 3′-Me, 4-DAB-carcinogenesis. Two upper curves relate to oxygen uptake of succinoxidase in the control (C) and experimental sample (E). Lower curves refer to anaerobic glycolysis in control and experimental.

TABLE V.—*Distribution of Cytoplasmic RNA during Carcinogenesis*

| Days | $\frac{\text{Non-sedimented}}{\text{sedimented}}$ RNA × $10^{-12}$ g Control | Experiment | Anaerobic glycolysis |
|---|---|---|---|
| 0 | $\frac{7\cdot4}{24\cdot0}=0\cdot3$ | — | — |
| 50 | $\frac{4\cdot6}{14\cdot0}=0\cdot33$ | $\frac{4\cdot2}{12\cdot0}=0\cdot35$ | 0 |
| 121 | $\frac{4\cdot0}{12\cdot0}=0\cdot33$ | $\frac{4\cdot8}{11\cdot0}=0\cdot44$ | 100% |
| 130 | $\frac{4\cdot6}{23\cdot0}=0\cdot202$ | $\frac{3\cdot9}{14\cdot4}=0\cdot27$ | 0 |
| 146 | — | $\frac{5\cdot5}{11\cdot0}=0\cdot5$ | 150% |
| 187 | $\frac{5\cdot5}{16\cdot2}=0\cdot31$ | $\frac{6\cdot0}{7\cdot3}=0\cdot82$ | 300% |
| 215 | — | $\frac{7\cdot3}{8\cdot9}=0\cdot84$ | 500% |
| Hepatoma (rat) | — | $\frac{10\cdot6}{10\cdot0}=1\cdot06$ | 1200% |
| Ehrlich ascites tumour | — | $\frac{12\cdot5}{2\cdot5}=5\cdot0$ | 3000% |
| Mouse sarcoma | — | $\frac{8\cdot7}{4\cdot0}=2\cdot2$ | — |
| Mouse mammary adeno Ca | — | $\frac{5\cdot4}{4\cdot2}=1\cdot3$ | — |

It can be seen that with the sudden increase of fermentation a change in distribution of cytoplasmic RNA takes place in the cell in such a way that the relative proportion between sedimented (ergastoplasm plus mitochondria) and non-sedimented RNA shifts in favour of the latter. In developed tumours this excess of non-sedimented over the sedimented RNA was still more striking. In the last columns of Table V three mouse tumours are also listed. It can be seen that they display the same or even greater preponderance of non-sedimented RNA over the sedimented cytoplasmic RNA fraction. The converse was also seen ; when on one occasion no increase of fermentation occurred despite 130 days of feeding with azodye, there was no increase in the relative proportion of the non-sedimented RNA fraction.

4. *Intracellular changes in levels of polarographically active sulphydryl groups*

The onset of increased anaerobic glycolysis characterizes thus an important turning point in the process of carcinogenesis. In an attempt to characterize further the two stages of carcinogenesis we studied the distribution of free, non-

FIG. 4.—Polarographic (anodic) waves of rat liver homogenates showing the drop in the level of GSH of the experimental sample.

protein, polarographically active, SH and SS groups. Fig. 4 illustrates the great reduction in the level of GSH in rat liver homogenate after 5 months of feeding with 3′-Me,4-DAB. This reduction is even greater in Novikoff hepatoma when compared with the GSH level at the onset of the carcinogenic diet.

The time changes in the levels of GSH during carcinogenesis are shown in Table VI. It may be seen that diet alone did not have any significant influence nor did the presence of carcinogen affect the level of GSH up to the period of approximately 80–100 days after the start of the feeding. After this period, however, a rather sudden drop in GSH levels per unit of DNA occurred. The developed liver tumours and Novikoff hepatoma contained only some 20 per cent of the normal liver GSH.

A completely different result was obtained when the total amount of non-protein sulphydryl groups was estimated polarographically by using the catalytic

TABLE VI.—*Polarographic Test for GSH in Homogenized Liver during Carcinogenesis*

| Tissue | Normal liver | Liver fed basal diet | | Liver fed 3′-Me-4-DAB | | | | | | | | Novikoff hepatoma |
|---|---|---|---|---|---|---|---|---|---|---|---|---|
| Time (days) | — | 30 | 180 | 30 | 60 | 80 | 100 | 110 | 150 | 180 | 210 | — |
| Value (cm.) | 10·8 (5 determ.) | 9·9 | 8·4 | 11·9 | 12·0 | 11·0 | 7·0 | 5·4 | 3·8 | 4·0 | 2·56 | 1·34 (5 determ.) |

These data refer to height in cm. of polarographic anodic wave 2GSH → GSSG + $H_2$ as shown directly in the homogenate containing approximately 100 × $10^6$ units of DNA in 1 c.cm.

effect of SH- (and SS-groups) on the dropping mercury electrode in the presence of ammoniacal solution of cobalt. When a solution of trivalent cobalt, which does not react with low molecular sulphydryl groups, was used, no significant difference was seen between controls and experimental samples, indicating that

FIG. 5.—Effect of carcinogenesis (60 days feeding 3′-Me-4-DAB) on free, polarographically active, sulphydryl groups in the supernatant of liver.

1. Control (basal diet)

2. Experimental

**1 and 2:** 0·2 ml. supernatant (dilution 1 : 10) added to Brdička's solution containing trivalent cobalt ($Co(NH_3)_6Cl_3$. No difference in polarographically detectable SH-content of soluble proteins.

3. Control (basal diet)

4. Experimental

**3 and 4:** *Idem* but Brdička's solution contains bivalent cobalt ($CoCl_2$). Experimental sample shows increase of non-protein SH-group.

5. Control (basal diet)

6. Experimental

**5 and 6:** Effect remains in deproteinized (sulfosalicylic acid) samples.

the amount of proteoses (or possibly also mucoproteins) is approximately the same in both cases. On the other hand a striking effect was seen in experimental samples when the same test was performed using bivalent cobalt. In this case the catalytic current at the cathode showed a much higher catalytic wave in the experimental sample than in the control, regardless whether the test was done with liver a month after start of carcinogen feeding or with tissue containing gross tumours. An illustration of this test is shown in Fig. 5.

The height of the catalytic SH-wave of the deproteinized supernatant was seen to increase very soon after the introduction of carcinogen. This effect is the more striking as, on the whole, the protein content of this fraction lags behind controls in the absolute amount. The effect disappeared for the most part when the supernatant was preincubated with monoiodoacetic acid (4 mg. in total volume of 3 ml.) at slightly alkaline pH for 30 minutes. The increased catalytic current at the cathode is undoubtedly due to the increased total level of sulphydryl (SH, not SS) groups as the result of carcinogen feeding.

5. *Proliferation and the onset of malignancy during carcinogenesis in liver*

As shown in the preceding sections there occurs during carcinogenesis a gradual depletion of cytoplasmic structures. The results represent only a statistical picture of an effect on a heterogenous population of several kinds of cells from which the liver consists. It might be that already in the first stage before the increased glycolysis appears; many of the parenchymatous liver cells die and are replaced by cells poor in cytoplasmic structures, *e.g.* by cells of proliferating biliary ducts. On the other hand it might be also that the dedifferentiation is not necessarily due to the inability of the cell to replace the existing subcellular elements damaged by a carcinogen in a numerically unchanged population of cells, but may result from premature divisions induced by the carcinogen before the cytoplasmic content of cells and their volumes doubled (Needham, 1952 ; Berglas, 1957).

One may assume that if either of these explanations fitted the actual course of events, the cell population during carcinogenesis would not remain numerically constant but would undergo very conspicuous variation. On the other hand if no such variation for a given period of time is observed, one has to assume that the dedifferentiation described and so easily detected by differential centrifugation is due to processes occurring within a great majority of the cells in relatively stable cell population. The process of reparation and proliferation, although conspicuous to a pathologist by histological methods, could apply only to a minority of the cell population of the whole organ which could not account for gross cytoplasmic depletions detected by differential centrifugation.

Table VII correlates the total amount of DNA in liver (expressed in DNA units) with liver weight during the whole period of carcinogenesis. It was seen that whereas liver weight is subject to great fluctuation, the DNA amount is practically constant for at least 75 days, that is, for the period of time during which gross changes in cellular composition, from the viewpoint of differential centrifugation, took place.

A massive proliferation of cells was detected only in the second period of carcinogenesis, after or simultaneously with the increased glycolysis. The relatively stable cell population during first stage of liver carcinogenesis indicates, therefore, that the depletion of cytoplasmic structures in the organ does not stem to any greater extent from the replacements of one cell type by another nor from premature cell divisions but mostly from a process affecting the bulk of the organ.

### DISCUSSION

The data obtained seem to establish two distinct stages in the process of liver carcinogenesis induced by 3′-Me, 4-DAB. In the first stage, which takes about half

TABLE VII.—*Liver Weight and Cell Proliferation during 3′-Me, 4-DAB Carcinogenesis*

| Days | | 0 | 11 | 19 | 21 | 44 | 50 | 64 | 71 | 75 | 100 | 119 | 192 |
|---|---|---|---|---|---|---|---|---|---|---|---|---|---|
| g Liver weight | C | 6·85 | — | 3·20 | 5·6 | 5·56 | 4·73 | 4·2 | 8·5 | 5·5 | 6·0 | 6·85 | — |
| | E | — | 6·6 | 5·39 | 5·65 | 3·8 | 6·25 | 5·3 | 7·68 | 6·5 | 9·28 | 18·3 | 11·85 |
| % Body weight | C | 4·16 | — | 2·7 | 2·9 | 4·5 | 3·2 | 3·5 | 3·3 | 2·8 | 3·3 | 3·3 | — |
| | E | — | 4·2 | 3·7 | 4·0 | 3·3 | 4·4 | 4·2 | 4·2 | 3·9 | 5·8 | 8·0 | 7·3 |
| DNA units $\times 10^6$ | C | 3,200 | — | 3,175 | 3,426 | 3,100 | 3,100 | 2,340 | 3,260 | 3,596 | 3,300 | 3,000 | — |
| | E | — | 3,380 | 4,140 | 3,400 | 3,100 | 3,071 | 3,230 | 3,880 | 3,819 | 6,000 | 13,730 | 5,250 |
| Anaer. glycog.* | C | — | — | — | — | — | — | — | — | — | — | — | — |
| | E | 0 | 0 | 0 | 0 | 0 | 0 | 0 | +20% | +50% | +100% | +200% | +250% |

* Excess over the control.

*Note* : Animals for this experiment were chosen with regard only to length of feeding regardless of body weight.

of the time span of the whole process, the cell suffers respiratory damage due to the gradual depletion of mitochondria and organized ergastoplasm. The carcinogen is not a respiratory inhibitor and the respiratory damage is, therefore, only a secondary effect of carcinogen binding.

The second stage is characterized not only by a relatively sudden increase of glycolysis supplementing the insufficient respiration but also by a sudden change in the distribution of non-protein sulphydryl groups and, finally, by a redistribution of cytoplasmic ribonucleic acid. The circumstance that all these changes occur — within the limits of observation — at the same time, is apparantly significant. If we look upon the animal cell as an Homeostat (Fiala, 1958) we may say that these variables behave as step functions suddenly changing their values when the system reaches its critical state (Ross Ashby, 1952). By entering a new equilibrium, marking malignant transformation, the cell solves its problem of adaptation.

The parallelity of effects on mitochondria and ergastoplasm obtained by application of anterior pituitary hormones and by application of carcinogen suggests that both these cytoplasmic structures depend on a common factor which is influenced in opposite ways by hormone and by carcinogen.

The basic fact, from which all observations reported in this work originated, was the scarcity of mitochondria in liver tumours. The question must, therefore, arise : Can the carcinogenic process be considered as resulting always from the depletion of mitochondria and ergastoplasm so that the observations here reported may have general validity or are they pertinent only to liver carcinogenesis ? It has been reported (Mateyko and Kopac, 1956) that ovarian tumours are marked by a negligible amount of peripheral cytoplasm, as compared with normal ovarian controls, which themselves have relatively little cytoplasm. Even in the giant Sternberg cells of Hodgkin's lymphoma the cytoplasm contains very few mitochondria and little of the endoplasmic reticulum (ergastoplasm) as observed by electron microscopy (Frajola *et al.*, 1956) ; in contrast enormous multilobal nuclei are present. A striking depletion of cytoplasmic structures in precancerous and cancerous skin was also observed with electron microscopy (Albertini, 1952,

1953). The depletion of mitochondria and of ergastoplasm is thus a characteristic feature of tumour cells.

It has been argued (Laird, 1954) that in certain tissues such as pancreas or lymph nodes the amount of mitochondria is also low and that it is incorrect, therefore, to ascribe any importance to the small quantity of mitochondria in tumours. This criticism is invalid because it is meaningless to compare pancreas with hepatoma ; pancreas should be compared with pancreatic tumour and lymph nodes only with lymphoma. Similarly it has been claimed (Schmidt and Schlief, 1955) that the cells of Ehrlich ascites tumour contain a large amount of porphyrins and cytochromes and demonstrate high $Q_{O_2}$-value. We have demonstrated, however, that the respiration is actually very low when expressed on the basis of DNA units (Table I). Also the fractionation of these cells (Fiala, 1958*b*) convinced us that in this tumour the mitochondria are scarce in proportion to DNA content.

In special instances it may be wrong to assume that the homologous normal tissue is the adequate control of a tumour. Thus by comparing the respiration of normal mouse epidermis with skin tumours it was concluded (Carruthers and Suntzeff, 1947) that tumour growth leads to an increase of succinoxidase activity and a greater respiration. Notwithstanding the fact that any comparison must be done on a per cell basis, or, still better, on a unit of DNA, one must point out that all the normal epidermal cells are not the natural counterpart of the skin tumour. Much of the epidermis consists of very differentiated cells the cytoplasm of which is almost completely filled out with tonofibrils with only few remaining preformed cytoplasmic elements. These cells are keratinized and, consequently, have a low respiration. There the loss of metabolic function is the result of normal differentiation in order to assume a specialized protective function. Skin tumours, on the other hand, stem from the cells which preceded normal differentiation. It is from the basal cells that both normal differentiation and malignant transformation stem and with which the only valid comparison can be made. It seems, thus, that all main objections against the thesis of mitochondrial depletion in tumours can be answered satisfactorily.

In contrast to our finding of sudden increase in anaerobic glycolysis, Nakatani *et al.* (1936) observed this increase right after the initiation of carcinogen feeding. These findings were not confirmed by Orr and Stickland (1941) but their data do not seem convincing either on the ground of Burk's criticisms (Burk, 1942). The data of Nakatani can probably be explained by the circumstance that this early investigator could not express his determinations on a per cell or unit of DNA basis. In consequence the anaerobic glycolysis of tissue depleted in weight by the diet but unchanged in regard to the number of cells could appear as having increased per unit of weight.

Our experiments did not reveal the nature of this increase of glycolysis in tumours. But it is well known that under appropriate conditions and in the presence of certain fortifying factors the homogenates of the normal tissue may ferment as well or even more vigorously than the homogenates of tumours (Meyerhof, 1949 ; Le Page, 1950). It seems, therefore, that the high fermentation of tumours is the reflection of some change in the regulation of intracellular metabolic processes rather than of the general increase in the level of glycolytic enzymes. The circumstance of increased anaerobic glycolysis coinciding with massive proliferation of the cells suggests that further increase in glycolysis is due only to the increase in the number of malignant cells. In other words in each of these cells the metabolic change is complete and maximal. This would allow to estimate the number of malignant cells present in a given piece of tissue simply from the rate of anaerobic glycolysis and DNA content.

In conclusion one may say that there can hardly be any doubt that the respiratory damage of the tumor cell is well established. However, inasmuch as the carcinogen does not induce the respiratory damage directly, the mechanism of carcinogenesis needs further elucidation.

SUMMARY

1. Experimental evidence was given to show that the carcinogenic compounds 3′-methyl,4-dimethyl aminoazobenzene and 3,4 benzpyrene are not respiratory inhibitors, although their accumulation inside the cell leads to a marked lowering of respiration, especially when the respiration is expressed per unit of DNA.

2. The respiratory damage is due to the reduction in the amount of mitochondria per cell.

3. Mitochondria and the ergastoplasm-bound RNA are regulated by a common factor which in the liver is influenced by a carcinogen (3′-Me, 4-DAB) in a way opposite to the action of anterior pituitary hormones in their target organs.

4. There is a critical period at which the anaerobic glycolysis suddenly increases, a shift in the distribution of cytoplasmic ribonucleic acid takes place in favour of the unorganized supernatant form and a shift in the protein-free polarographically active sulphydryl groups is observed.

5. This critical period marks the massive proliferation of cells.

The senior author (S.F.) wishes to thank Dr. Edith E. Sproul, Department of Pathology, Columbia University, for her interest in this work, which was aided by a grant (C-2441) from the National Institute of Health, U.S. Public Health Service, and also by a grant of the American Cancer Society.

Parts of this work were presented in abstracts at the Annual Meetings of the American Association for Cancer Research in Atlantic City, 1956 and at the Federation Meetings in Chicago, 1957.

REFERENCES

Albertini, A. v.—(1952) Schweiz. A. f. allg. Path. Bakt.; **15**, 452.—(1953) *J. nat. Cancer. Inst.*, **13**, 1473.

Allard, Cl., Lamirande, G. de, Cantero, A.—(1953) *Canad. J. med. Sci.*, **31**, 103.

Barker, S. B. and Summerson, W. H.—(1941) *J. biol. Chem.*, **138**, 535.

Berglas, A.—(1957) ‘ Cancer, Nature, Cause and Cure ’, Paris (A. Berglas, Ed.).

Brdička, R.—(1933) *Coll. Trav. chim. Tchécosl.*, **5**, 112.

Burk, D.—(1942) in ‘ A Symposium on Respiratory Enzymes ’, p. 235 (University of Wisconsin Press).

Carruthers, Ch. and Suntzeff, V.—(1947) *Cancer Res.*, **7**, 9.

Davidson, J. N. and Leslie, I.—(1950) *Nature, Lond.*, **165**, 49.

Fiala, S.—(1953) *Naturwissenschaften*, **40**, 391.—(1958*a*) Abstr. *IV. int. Congr. Biochem., Vienna* (Section 6, No. 31).—(1958*b*) *Naturwissenschaften*, **45**, 366.

*Idem*, Sproul, E. E. and Fiala, A. E.—(1956) *J. biophys. bioch. Cytol.*, **2**, 115.—(1957) *Proc. Soc. exp. Biol., N.Y.*, **94**, 517.

FIESER, L. F.—(1941) ' Experiments in Organic Chemistry ', 2nd. Ed., New York and London (D. C. Heath & Co.).
FRAJOLA, W., GREIDER, N., BELLIOS, N. AND BOURNOCLE, B.—(1956) *Proc. Amer. Ass. Cancer Res.*, **2**, 165.
GREENBERG, D.—(1955) *Cancer Res.*, **14**, 421.
HOŠČÁLKOVÁ, Z., ČERNOCH, M. AND ŠANTAVÝ, F.—(1955) *Coll. Trav. chim. Tchécosl.* **20**, 1096.
HOWATSON, A. F. AND HAM, A. W.—(1955) *Cancer Res.*, **15**, 62.
KOLTHOFF, I. M. AND LINGANE, J. J.—(1952) ' Polarography ', 2nd Ed., New York and London (Interscience).
LAGERSTEDT, S.—(1949) *Acta anat.*, Suppl. 9.
LAIRD, A. K.—(1954) *Exp. Cell Res.*, **6**, 31.
LAITINEN, H. AND SULLIVAN, B.—(1941) *Cereal Chem.*, **18**, 60.
LEPAGE, C. A.—(1950) *Cancer Res.*, **10**, 77.
MATEYKO, G. M. AND KOPAC, M. J.—(1956) *Proc. Amer. Ass. Cancer Res.*, **2**, 131.
MEYERHOF, O.—(1949), *Arch. Biochem.*, **23**, 246.
MILLER, E. C., MILLER, J. A., KLINE, B. E. AND RUSCH, H. P.—(1948) *J. exp. Med.*, **80**, 81.
MUNTWYLER, E., SEIFTER, S. AND HARKNESS, D. N.—(1950) *J. biol. Chem.*, **184**, 181.
NAKATANI, M., NAKANA, K. AND OHARO, Y.—(1938) *Gann*, **32**, 240.
NEEDHAM, A. E.—(1952) ' Regeneration and Wound Healing ', London (Methuen & Co.).
ORR, J. W. AND STICKLAND, L. H.—(1941) *Biochem. J.*, **48**, 33.
ROSS ASHBY, W.—(1952) ' Design for a Brain ', New York (J. Wiley).
SCHMIDT, G. G. AND SCHLIEF, H.—(1955) *Naturwissenschaften*, **42**, 105.
SCHNEIDER, W. C.—(1945) *J. biol. Chem.*, **161**, 293.
*Idem*, Hogeboom, G., Sheldon, M. and Striebich, M. J.—(1953) *Cancer Res.*, **13**, 285.
STADIE, W. C., RIGGS, B. C. AND HAUGAARD, N.—(1945) *J. biol. Chem.*, **160**, 191.
THOMSON, R. Y., HEAGY, F. C., HUTCHISON, W. C. AND DAVIDSON, J. N.—(1953) *Biochem. J.*, **53**, 260.
VENDRELY, R.—(1955) in ' The Nucleic Acids ', Vol. 2., pp. 155–180, New York & London (Academic Press).
WARBURG, O.—(1926) ' Ueber den Stoffwechsel der Tumoren ', Berlin, (J. Springer).—(1956) *Science*, **123**, 309.
*Idem*, BURK, D. AND SCHADE, A. L.—(1956) *Ibid.*, **124**, 267.
WEINHOUSE, S.—(1951) *Cancer Res.*, **11**, 585.—(1956) *Ibid.*, **15**, 654.—(1956) *Science*, **124**, 267.
*Idem*, MILLINGTON, R. H. AND WENNER, C. E.—(1951) *Cancer Res.*, **11**, 845.
WENNER, C. E., SPIRTES, M. A. AND WEINHOUSE, S.—(1951) *Proc. Soc. exp. Biol., N.Y.*, **78**, 416.